# Heat Shock Protein: Hard Worker or Bad Offender for Gastric Diseases

**DOI:** 10.1155/2010/259163

**Published:** 2010-09-29

**Authors:** Ho-Jae Lee, Chan Young Ock, Seong-Jin Kim, Ki-Baik Hahm

**Affiliations:** ^1^Laboratory of Chemoprevention, Lee Gil Ya Cancer and Diabetes Institute, Gachon University of Medicine and Science, Incheon, Republic of Korea; ^2^Laboratory of Translational Medicine, Lee Gil Ya Cancer and Diabetes Institute, Gachon University of Medicine and Science, Incheon, Republic of Korea; ^3^Laboratory of Cell Regulation and Carcinogenesis, Lee Gil Ya Cancer and Diabetes Institute, Gachon University of Medicine and Science, Incheon, Republic of Korea; ^4^Department of Gastroenterology, Gachon Graduate School of Medicine Gil Medical Center, Songdo-dong 7-45, Yeonsu-gu, Incheon, 406-840, Republic of Korea

## Abstract

Heat shock proteins (HSPs) have core housekeeping functions in the cells where they are built-in components of folding, signal transduction pathways, and quality control functions for which they proofread the structure of proteins and repair misfolded conformers. *Helicobacter pylori* (*H. pylori*) infection leads to significant inflammations in the gastric mucosa, which is closely associated with development of either precancerous lesion including chronic atrophic gastritis or gastric cancer in addition to, peptic ulcer disease, and mucosa-associated lymphoid tissue (MALT) lymphoma. Therefore, the association between *H. pylori* infection and role of HSP has been focused as an important issue because there had been rather conflicting publications showing that HSPs as a good worker for defense against *H. pylori* infection, whereas HSPs as a bad offender contributing to the progression of *H. pylori*-associated gastric carcinogenesis in addition to aggravation of gastric inflammation. In this paper regarding proteomic discovery of HSPs related to *H. pylori*-associated gastric diseases, we introduce several evidences obtained from proteomic analysis dealing with friend or foe role of HSP in *H. pylori* infection from a cellular level to human diseases. The implication of HSPs in alcoholic or NSAIDs-induced gastritis and the intervening of HSPs in biological changes exemplified with TGF-*β* signaling, key tumor suppressor growth factors regulating inflammation, immune function, and carcinogenesis were further introduced.

## 1. Introduction

Epithelial cells in gastrointestinal (GI) tract are continuously exposed to a lot of stressful antigens because we eat different meals 3 times a day and swallow air pollutant into GI tract. Moreover, they are harassed to several kinds of gas produced by commensal bacteria colonized in GI tract. To cope with this very stressful organization and environment, GI mucosa wisely developed various strategies to tolerate stresses, such as generating antioxidant enzymes, increasing level of immune-tolerance, and regulating mechanisms to cope with stress like chaperone genes including heat shock proteins (HSPs). HSPs are groups of stress-response proteins which are either constitutively expressed or induced through the transcriptional action of heat shock factor (HSF). HSPs are classified into four major families according to their biological activities and apparent molecular weights; HSP90, HSP70, HSP60, and small HSPs including HSP27. While HSP60, HPS70, and HSP90 are constitutively expressed, HSP70 and HSP27 are induced by various conditions, including heat, oxidative stress, or drug exposure. The type of HSP induced and its level of expression can determine the fate of a cell in response to stress or stimulus, by which HSPs may play a cytoprotective role in gastrointestinal tract. For instances, oral administration of geranylgeranylacetone (GGA), an antiulcer drug, rapidly induced HSP70 in rat gastric mucosal cells and the induced HSPs contributed to the suppression of inflammation accompanied with accelerated healing of ulcer induced by water immersion restraint stress. Animal studies have consistently demonstrated that though *H. pylori* infection delays gastric mucosal healing by disrupting the balance in cell apoptosis and proliferation, decreasing migration of epithelial cells, and decreasing blood flow and angiogenesis within the gastric mucosa, HSPs could reverse these limitation and inferiorities in mucosal healing. 

The general roles of HSP are intracellular cleaning of wasted product due to stress, after which are nominated as molecular chaperone, of which functions are as follows briefly: (1) assisting folding of translated proteins, (2) importing proteins into subcellular compartment, (3) disassembling oligomeric protein structures, (4) inducing proteolytic degradation of unstable proteins, (5) controlling the biological activity of folded regulatory proteins, and (6) endogenous modulation of apoptosis execution [1–4]. Interestingly in contrast to these protective roles for mammalian cells, HSPs could facilitate cell damage and promote carcinogenesis. Moreover, increasing evidences showed that not only mammalian cells have HSPs, but also bacteria such as *Helicobacter pylori* (*H. pylori*) have HSPs either to survive against hostile host offense system or to disrupt host defense system. 

Therefore, this bifunctional significance of HSPs as “worker” based on contribution to the strengthening gastric defense system or “offender” based on weakening gastric defense system besides of molecular mimicry prone to autoimmune in gastric diseases is one of intriguing issues in gastroenterology. Here in this paper, we present double-edged roles of HSPs in *H. pylori* or alcohol-associated gastritis, followed with the novel strategy that HSPs can be a target for treatment or diagnostic biomarker for predicting the progression of gastric disease all documented with proteomic approach. Brief workflow for proteomic approach will also be introduced before HSP-related research in gastric diseases.

## 2. Workflow for Proteomic BiomarkerDiscovery of Gastric Diseases

Every patient, even diagnosed with same disease, has different profile of gene or protein expression, a very important starting point of view for tailored medicine. To find out the common profile of a subset of patients with same disease is one of challenging issues from late 1990s. Using genomic data originated from Human Genome Project (HGP), we could reload a powerful bullet in our hands to analyze the similarity or difference of gene expression profile of patients, after which it has not taken long to find out that there were a lot more regulation above the simple sequence of genes and their expression such as epigenetic regulation or posttranslational regulation. Conclusively, “proteomics” approach including 2D-PAGE and mass spectrometry is indispensible in order to understand global protein expression and modification profile. Using these cutting edge techniques, we could understand the whole protein status at a glance instead of old-fashioned technique of molecular biology which would have consumed a lot more time and efforts and touched the modification of whole protein in a rather easy way [5, 6]. One of very useful applications of proteomics is developing clinical biomarker to predict the existence or severity of diseases.

Potential biomarkers would be suggested by comparing the protein profile of patients with that of normal people, after which we could verify the significance of this biomarker by analyzing large-scaled clinical study. The biologic role of this potential biomarker would be validated by conventional molecular biological technique. If the quantitative level of potential biomarker correlates well with severity of disease or progression of inflammation-carcinogenesis sequence, this marker is not only useful in clinical screening but also in studying pathogenesis of disease. Therefore, proteomics research has been developed as a fancy bridge from bench to bed side, especially in understanding diseases which have “inflammation-carcinogenesis” sequence [7].

**Figure 1:**
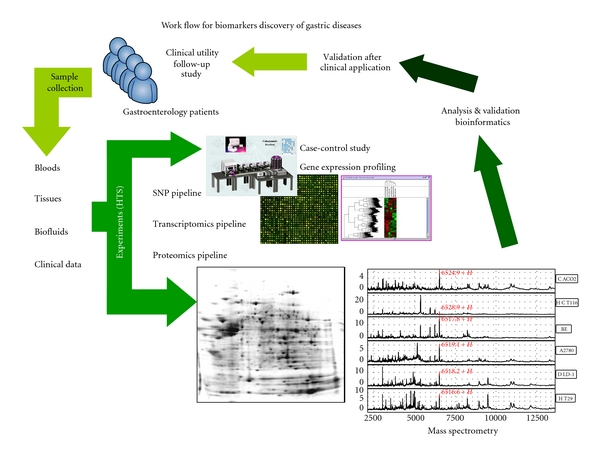
*Workflow for discovering potential biomarkers of gastric diseases.* HGP contributed to development of either the concept of high-throughput analysis or pipeline for experimental technology, including SNP discovery, transcriptomics, proteomics, metabolomics, clinomics, and glycomics. Proteomic pipeline for serum biomarker discovery, pathogenic proteins elucidation, and precision medicine is actively working for tailored medicine.

Before the era of “omics,” clinician diagnosed the patient with his/her own clinical impression based on medical history and physical examination. This basic technique of “taking care of” patient was the most fundamental, but very important manner to a clinician. However, as increasing level of understanding pathophysiology of diseases and as increasing capacity of clinical data, this trial-error approach or experience-based manner shows the limitation in individualization of patient care, necessitating the need of “dissecting” his/her biologic profiles with the technique of genomics, transcriptomics, proteomics, and metabolomics. After routine history taking and physical examination, patient samples such as bloods, tissues, or biofluids would be gathered in gastroenterology clinics and would be transferred to 3 different pipelines, comprising of single nucleotide polymorphism (SNP) pipeline, transcriptomics pipeline, and proteomics pipeline. Though case-control study is one example of clinical study methods using analyzing correlation between disease status and SNP status, whether SNP is different between patients' and controls' could not confirm that it affects real biologic difference because SNP may not involve in transcriptional regulation. We could solve this problem to analyzing whole transcriptional regulations by microarray or protein-array methods, so-called “transcriptomics.” However, soon beyond-transcriptional regulation became more important in biologic consequences. Post-translational modification such as phosphorylation, glycosylation, protein folding, protein trafficking, and protein-protein interaction would be caught by proteomics pipeline. This pipeline includes 2-dimensional electrophoresis and mass spectrometry. Using these three kinds of pipeline, we could gather amount of information about patients' biologic status. These abundant, ambiguous, and sometimes nonspecific data will be analyzed and validated by statistical significance, after which data will be compared with clinical outcomes. At last using this workflow, we could understand the important pathophysiologic mechanism of diseases, and suggest more validated strategy to individual patient due to statistical evidence (Figure 1).

## 3. Host Heat Shock Protein (HSP) in *H. pylori* Infection: Roles as Hard Worker or Bad Offender

### 3.1. HSP as Hard Worker

Gastric surface mucous cells are the first line of defense against insults like ingested foods, nonsteroidal anti-inflammatory drugs (NSAIDs), alcohol, and *H. pylori* infection [8], within which HSPs are crucial for the maintenance of epithelial homeostasis during normal cell growth and for survival during and after various cellular stresses, supported with their molecular chaperone and cytoprotective actions like either protecting mitochondria or interfering with the stress-induced apoptotic programme [9]. In order to document the contribution of HSPs after *H. pylori* infection, first we performed two-dimensional electrophoretic analysis in order to check any shifts in HSP profiles after *H. pylori* infection [10]. As results, *H. pylori* infection significantly attenuated the expression of HSP70 whereas exposure of cells to noncytotoxic heat shock or geranylgeranylacetone (GGA), an HSP inducer, restored HSP70 expression, as well as suppressing the expression of iNOS, a major inflammatory mediators provoking *H. pylori*-induced gastric tissue damage. Our results suggest that induction of HSP70 confers cytoprotection against *H. pylori* infection by inhibiting the expression of iNOS. These results provided important insights into the flux in HSPs profiles in response to *H. pylori* infection and highlighted the cytoprotective role of HSP70 in *H. pylori* infection. Since paradoxically *H. pylori* have been found to decrease expression of HSPs, Axsen et al. [11] investigated whether this phenomenon of HSP downregulation is specific to *H. pylori* or not. As results, coculture of *H. pylori* with two gastric carcinoma cell lines reduced expression of HSP70 and, to a lesser extent, HSP60. Downmodulation of HSPs was not dependent on the presence of the vacuolating cytotoxin (*vac*A) or the *cag* pathogenicity island (*cag* PAI) whereas the bacterial pathogen, *S. typhimurium*, upregulated HSP expression reversely. Therefore, though sometimes HSPs are thought to function as danger signals during microbial infection, *H. pylori*-induced downregulation of HSPs could be a mechanism of immune evasion that promotes chronic infection [12]. 

**Figure 2:**
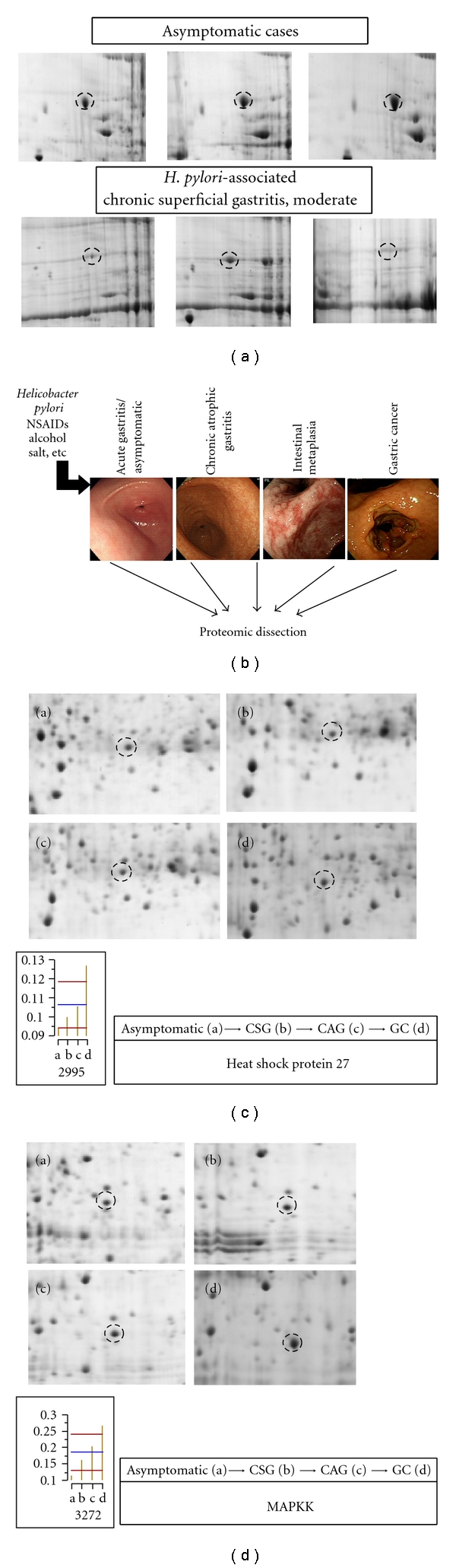
*Proteomic dissection for potential biomarkers for disease progression and carcinogenesis.* (a) Helicobacter pylori-associated chronic superficial gastritis was significantly associated with attenuated levels of HSP70 as identified in previous publication [10]. (b) Biopsied tissues obtained from diverse stage of *H. pylori*-associated gastric diseases were used for proteomic analysis. (c) On individual analysis after (b), significant correlation was noted between the increased spot density of HSP27 and the progression of *H. pylori*-associated gastric disease, confirming previous publications that HSP27 can be one of potential biomarker for *H. pylori*-associated gastric diseases. (d) MAPKK can be another candidate telling the progression of *H. pylori*-associated gastric diseases.

Next, in order to verify these attenuations of HSP70, documented as “hard worker” for enhancing gastric mucosal defense, after *H. pylori* infection, we have compared the proteomes between *H. pylori*-not-associated asymptomatic stomach and *H. pylori*-associated chronic gastritis. Very interestingly, one of significant changes was “decreased HSP70 level” in *H. pylori*-associated chronic superficial gastritis patients (Figure 2(a)), similar finding as noted in the above cellular system. As mentioned above, since HSP70 proteins assist a wide range of folding processes, including the folding and assembly of newly synthesized proteins, refolding of misfolded and aggregated proteins, membrane translocation of organellar and secretory proteins, and control of the activity of regulatory proteins [9], HSP70 proteins have additional housekeeping functions in balancing cellular homeostasis. Decreased HSP70 expression in *H. pylori* infection has been reported in gastric epithelial cell line [13] and *in vivo* animal studies [14]. In conclusion, HSPs are finely regulated in a response to various extracellular stresses and reversely the disruption of HSPs regulation might result in fatal consequence of gastric disease status. These biologic meanings significantly correlated with clinical outcome strongly suggested that HSPs are “hard worker” for gastric defense. Then, curiosity arose that how about the changes of other subfamily of HSPs, for instance, HSP90 or HSP27 in *H. pylori* infection? Since HSP90 has been revealed to be critical for intracellular signaling that participates in inflammatory response as well as carcinogenesis [14], we have investigated a regulatory role of HSP 90 in *H. pylori*-induced IL-8 production [15], showing that *H. pylori* stimulated significant phosphorylation of HSP90, but the phosphorylation was diminished by administration of HSP 90 inhibitor, geldanamycin (GA). Treatment of GA completely inhibited *H. pylori*-induced IL-8 production through the deactivation of ERK1/2 and NF-*κ*B. These results subsequently lead to inactivation of AP-1 and NF-*κ*B, which are known to be major transcriptional factors of IL-8. Our investigation provides important insights that HSP90 is involved as a crucial regulator in the production of *H. pylori*-induced IL-8 chemokine and HSP90 inhibitor could be potentially used for the inhibition of *H. pylori*-provoked inflammation. Though HSPs seemed to be “hard worker” for gastric defense, reckless induction of HSPs can confront unwanted disaster, leaving the precept “much is not always good.”

### 3.2. HSP as Bad Offender

Reversely to “hard worker” role of HSPs, exposure of cells to microbial pathogens also induced HSPs, which then modulated both innate and adaptive immune responses, led to “bad offender” to perpetuating gastric inflammation or inducing autoimmune gastritis [16]. *H. pylori* infection induces autoantibodies that cross-react with human gastric mucosa from infected individuals. Candidates for the antigens responsible for molecular mimicry causing autoreactivity include the HspB (HSP60, sometimes called HSP54) or Lewis *x* and Lewis *y* carbohydrate antigens. Pierzchalski et al. [12] determined whether apoptosis induced in the gastric epithelium exposed to live *H. pylori* might occur due to either the elimination of HSP70 expression or deregulation of the heat shock response of the cell. What they have elucidated was that *H. pylori* infection induces autoantibodies that cross-react with human gastric mucosa from infected individuals. Infection with *H. pylori* induces humoral immune responses against various antigens of the bacterium, among which HSPs are immunodominant antigens. All of these results suggested that HSPs contributed to *H. pylori* colonization, facilitated mucosal infection, and promoted inflammation. Therefore, under the hypothesis that proteomic approach can provide the insights enable to pull out biomarkers for predicting the progression of gastritis and risk factors for gastric carcinogenesis, we have checked and compared the pulled-out proteomes from asymptomatic cases, symptomatic chronic superficial gastritis (CSG), chronic atrophic gastritis (CAG) accompanied with intestinal metaplasia (IM), and gastric cancer (GC). Gastric inflammation is a well-known example of inflammation-carcinogenesis sequence (Figure 2(b)). In an analysis, to simply focused on finding proteins whose expression sequentially increases or decreases as disease progress from *H. pylori*-associated asymptomatic case to gastric cancer. As results, one of significant changes was serial increment of “HSP27” (Figure 2(c)) and “MAPKK” (Figure 2(d)) level, the mean levels of which were significantly increased as disease progress from asymptomatic, CSG, CAG, to gastric cancer. Generally, in response to extracellular stress such as heat, oxidative stress, or anticancer agents, the small HSPs and HSP70 are induced while HSP90 and HSP60 are constitutively expressed. HSP27 was reported to increase the antioxidant defense of mammalian cells by increasing the level of reduced glutathione, GSH, and decreasing reactive oxygen species (ROS) [5]. By the way, in spite of these protective effects against cellular stress, increased expressions of HSP27 were also reported in a variety of cancers, for which it was reported that HSP27 induces antiapoptotic activity [4, 17–19]. Therefore, step-by-step increasing expression of HSP27 in progression of gastritis to premalignant condition cancer may be explained by either insufficient protective effect or directly harmful effect of HSP27, all of these plausible mechanisms might be associated with the progression and deepening of gastric inflammation. Another other potential biomarker for these aggravations of gastritis was increase in MAPKK, core signal for inflammation cascade and carcinogenesis [20, 21].

Development of gastric mucosa associated lymphoid tissue (MALT) lymphoma is thought to be closely associated with host immune reactions to *H. pylori*. To investigate humoral immune responses in patients with MALT lymphoma to antigens shared by *H. pylori* and human gastric epithelial cells, Kawahara et al. [22] screened sera from *H. pylori* positive patients with MALT lymphoma and other gastroduodenal diseases, all associated with *H. pylori* infection. Immunoblotting of sera from patients with MALT lymphoma often detected a band with a molecular mass corresponding to HSP60, and both ELISA and immunoblotting showed elevated antibody titres to the recombinant human HSP60, leading to that conclusion that autoantibodies reactive with host gastric epithelial cells are often increased in MALT lymphoma, and Hsp60 is a major target antigen. Therefore, immune responses induced by immunological cross reactivity between *H. pylori* HspB and human HSP60 in gastric epithelium may be involved in the development of MALT lymphoma. Finally, HSP90 is a molecular chaperone whose association is required for the stability and function of multiple mutated, chimeric, and overexpressed signaling proteins that can promote the growth and survival of cancer cells since HSP90 client proteins include mutated p53, *Bcr-Abl*, *Raf*-1, Akt, HER2/*Neu* (*Erb*B2), and HIF-1*α* [23]. This is why HSP90 has been identified as a critical regulator of oncogenic protein and we have also published that *H. pylori* infection is also associated with increased activity of these oncogenic proteins in either inflammation perpetuation or proliferative activities.

## 4. Bacterial Heat Shock Protein: Role as Foe in *H. pylori * Infection

**Figure 3:**
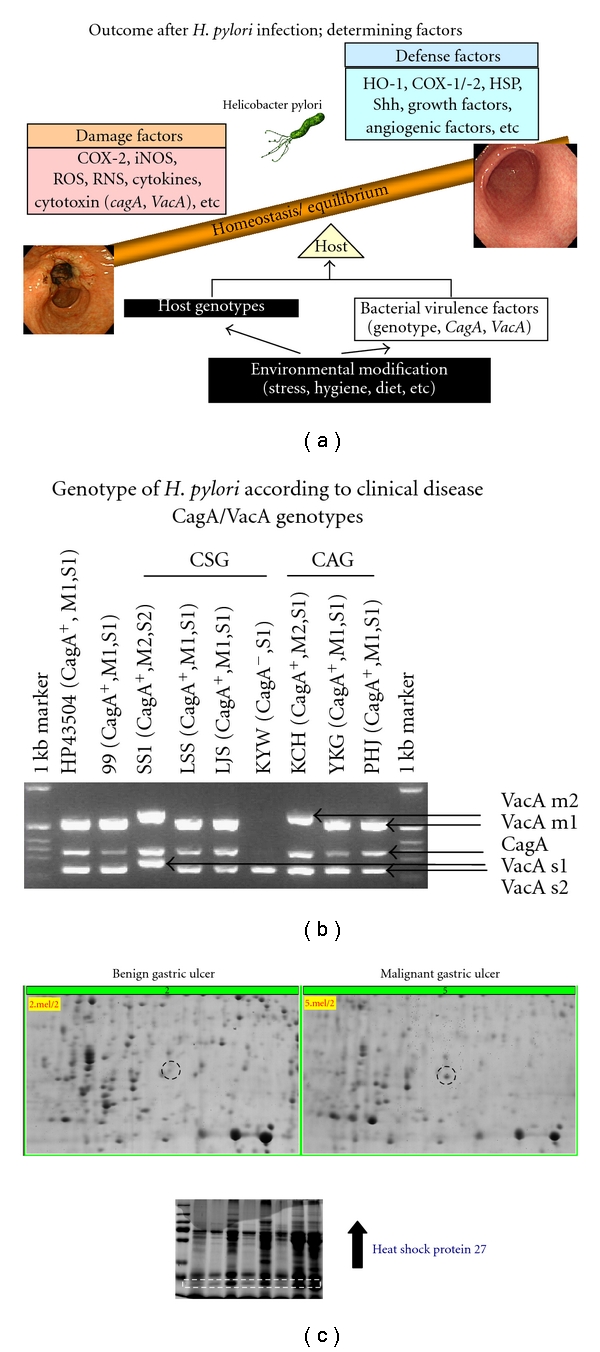
*Comparative proteomes of H. pylori related to gastric diseases.* (a) Bacterial virulence factor, host factor, and environmental factor might influence the outcome of *H. pylori* infection, disrupting the balance between damaging factor and defense factor. (b) Genotyping of *H. pylori* virulence gene according to isolates disease, CSG denotes *H. pylori* isolated from the patients with chronic superficial gastritis and CAG from patients with chronic atrophic gastritis. Though these virulence factors can affect the disease pathogenesis, they were not discriminative in differential diagnosis as biomarker. (c) Significantly increased spot of HSP27 were noted in *H. pylori* isolated from gastric cancer compared to benign gastric ulcer (lower panel). Using the sera obtained from the patient with gastric cancer, significant immunoblotting was noted in patients with gastric cancer, imposing the significance as biomarker for carcinogenesis.

Among *H. pylori*-infected patients, 80% remains asymptomatic along life whereas 10% suffered from associated disease including peptic ulcer and gastritis and less than 1% become the victim of gastric cancer [24], in which divergence host genetic factor, environmental influence, and bacterial virulence are known to be intercalated (Figure 3(a)). Although many *H. pylori* virulence factors have been reported to be pathogenic, we could not discriminately acknowledge those as potential biomarker. As shown in Figure 3(b), neither *cag*A nor *vac*A genotype was not correlated with clinical disease in spite of critical pathogenic contribution in our documentation in spite that the presence of *cag*A is associated with increased risk of gastric adenocarcinoma [25] and that statistical significance exists between bacterial genotype of CagA allele, VacA allele, and host disease status [26]. Lin et al. [27] studied to identify gastric cancer-related antigens from *H. pylori* using proteomic approach and characterize their roles in the development of gastric cancer. As results, the proteins showing higher frequency of recognition in *H. pylori*-associated gastric cancer group are as follows: threonine synthase, rod shape-determining protein, S-adenosylmethionine synthetase, peptide chain release factor 1, DNA-directed RNA polymerase alpha subunit, cochaperonin GroES (monomeric and dimeric forms), response regulator *Omp*R, and membrane fusion protein. In a similar approach, Lin et al. found a potential role of *H. pylori* HSP60 in gastric tumorigenesis. *H. pylori* HSP60 enhanced migration by gastric cancer cells and promoted tube formation by umbilical vein endothelial cells and triggered the initiation of carcinogenesis by inducing proinflammatory cytokine release and by promoting angiogenesis and metastasis. We also could find that *H. pylori* HSP27 was increased in bacteria cultured from mucosa of malignant gastric ulcer patient compared to bacteria obtained from mucosa of benign gastric ulcer patient, suggesting that bacterial HSP27 may have roles for bacterial survival or strength to attack host gastric mucosa, explaining functional similarity of mammalian HSPs (Figure 3(c)).

## 5. Host Heat Shock Protein inNon-*H. pylori*-Associated Gastritis

### 5.1. HSP in Alcoholic Gastritis

**Figure 4:**
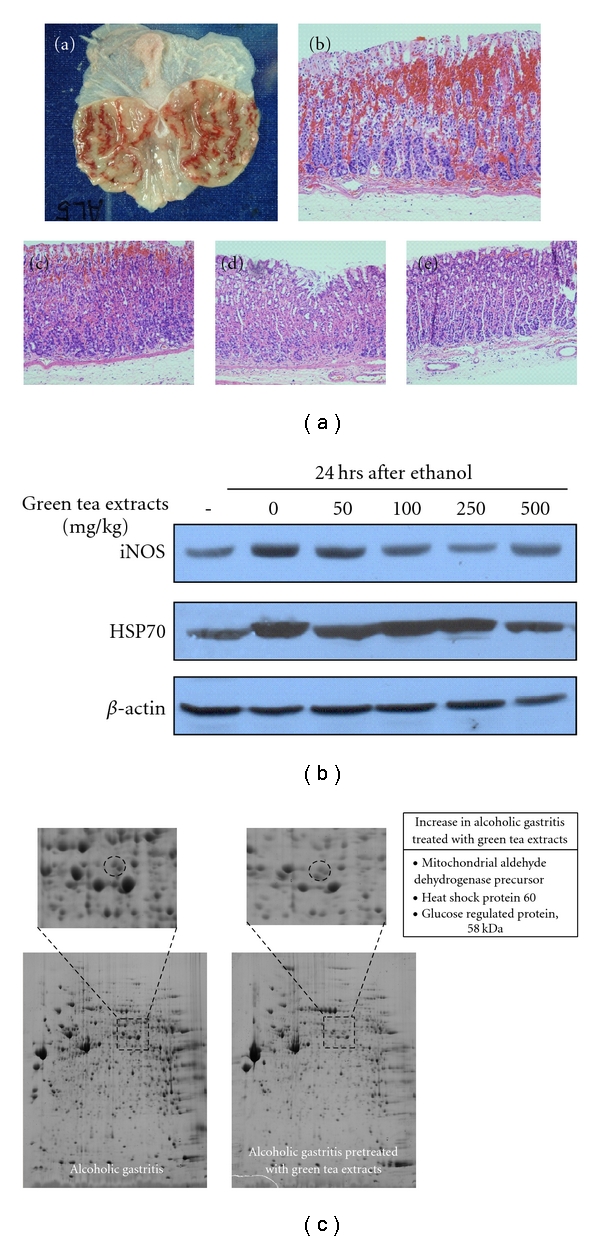
*Green tea-induced HSP for alcoholic gastritis.* (a) Intragastric administration of ethanol to male Sprague-Dawley rats caused significant gastric mucosal damage as shown gross and pathologic finding (a, b). Green tea extract, 50, 100, and 500 mg/kg, showed significant improvement in gastric pathology in a dose-dependent manner (c, d, e). (b) iNOS expressions were significantly decreased as the dosing of green tea extracts were increased whereas no significant change in HSP70 was observed on Western blotting of iNOS and HSP70 [36]. (c) The mucosal homogenates were subjected to 2D-PAGE and the spots were compared between alcoholic gastritis group and alcoholic gastritis pretreated with 500 mg/kg green tea extract. On mass spectroscopy, HSP60 and GRP58 were significantly preserved with increased acidic shifts.

Chronic alcohol consumption is highly associated with gastric and ulcer which may progress to gastric cancer. Alcohol-induced gastric mucosal injury can be mediated by various cellular molecules such as cyclooxygenase (COX), lipoxygenase (LOX), cytokines, and oxygen-derived free radicals [28–30] whereas polyphenols from green tea were reported to inhibit inflammation, scavenge excess free radicals, and stimulate the regeneration of damaged cells or tissues [31]. Based on these backgrounds, we hypothesized that green tea extracts may attenuate alcohol-induced gastric injury [32].  Figure 4(a) showed that the gross and pathology of alcohol-induced gastric damage and green tea extract pretreatment dramatically reduced gastric hemorrhage and mucoid cap formation in a dose-dependent manner. With green tea extracts, COX-2, iNOS levels were significantly decreased accompanied with inhibition of NF-*κ*1, and MAPK pathway. However, in contrary to our expectation, HSP70 level was unchanged even after green tea extract treatment (Figure 4(b)), necessitating further to confirm whether attenuating effect of green tea extract in alcoholic gastritis was not relevant to HSPs. To analyze the difference of protein profiles between alcohol-induced control and green tea extract pretreatment group, we performed proteomic analysis. Interestingly, we found HSP60 and glucose-regulated protein 58 (GRP58) levels were significantly increased in green tea extract, more apparent in the activation of these proteins as documented with acidic shift, suggesting that activation of HSPs with green tea was engaged in these protections from alcoholic gastritis rather than the levels of expression (Figure 4(c)). GRP58 is also a member of ER stress response genes, its role is mainly similar with HSPs' [33]. This inducible ER chaperone may be a diligent worker for coping with alcohol-induced cellular stress. Interestingly two opposite points of view regarding the role of HSPs also exists in alcoholic gastritis, imposing HSP as worker or offender in alcoholic damage. Kai et al. [34] studied the degree of gastric mucosal damage against 70% ethanol following GGA or vehicle treatment in portal hypertensive (PHT) rats because PHT gastropathy has an increased susceptibility to damage due to noxious factors. Ethanol-induced gastric mucosal damage was significantly decreased due to GGA treatment in the PHT rats, but not in the sham-operated rats, suggesting that HSP70 expression is enhanced in PHT gastric mucosa and plays an important role in gastric mucosal protection. On the other hand, Tominaga et al. [35] showed 10% ethanol pretreatment markedly increases gastric HSP90 expression in PHT rats. Excessive production of HSP90 may contribute to impaired adaptive cytoprotection.

### 5.2. Host Heat Shock Protein in NSAID-Induced Gastropathy

A major clinical problem encountered with the use of NSAIDs such as indomethacin is gastrointestinal complications. Both NSAID-dependent COX inhibition and gastric mucosal apoptosis are involved in NSAID-produced gastric lesions, and this apoptosis is mediated by the endoplasmic reticulum stress response and resulting activation of Bax. Since HSPs have been suggested to protect gastric mucosa from NSAID-induced lesions, we have tested the activation of HSPs are engaged in protection from indomethacin induced gastropathy using proteomic approach. NSAIDs were closely associated with increased phosphorylation of HSP27, after which the levels of HSP27 were significantly decreased through proteasomal degradation. Therefore, the way of preserving the HSP27 can be the one of efficient modalities to stomach free from NSAIDs damages irrespective of COX activity.

## 6. Translational View of HSP: As Exampled with Crosstalk between HSPs and TGF-*β* Signaling

**Figure 5:**
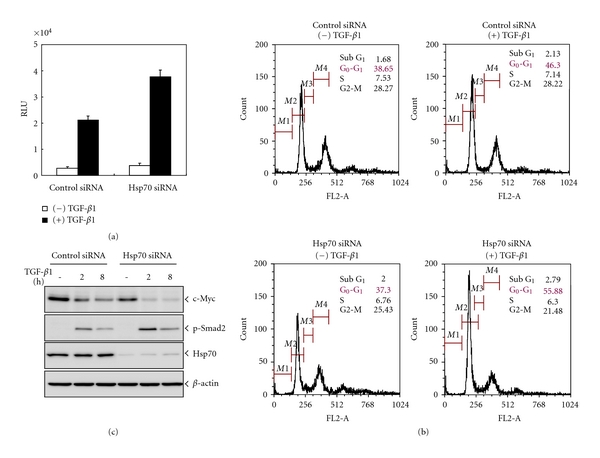
*Suppression of HSP70 sensitizes TGF- β* *signaling.* (a) NCI-H292 cells were transfected with HSP70 siRNA or control siRNA and SBE4-luc, and then treated with TGF-*β*1 (5 ng/ml) for 16 hr. Luciferase activity was measured by a luminometer. (b) Cells transfected with HSP70 siRNA or control siRNA treated with or without TGF-*β*1 (5 ng/ml) for 24 hr and stained with the PI DNA dye were subjected to cell cycle analysis using FACS. The data were expressed as a histogram with the DNA content (FL2) along the *X*-axis. (c) Cells were transfected with HSP70 siRNA or control siRNA treated with or without TGF-*β*1 (5 ng/ml) for indicated time. Cell lysates were immunoblotted with antibody specific to c-Myc, phospho-Smad2, or HSP70.

Among several kinds of HSPs, HSP90 is a ubiquitous molecular chaperone whose association is required for stability and function of many signaling proteins. HSP90 client proteins include a wide variety of signal transducing proteins that regulate cell growth and differentiation such as transcription factors, receptors, and protein kinases [37]. Recent studies show that TGF-*β* signaling mediators including TGF-*β* receptors, receptor-activated Smads, or inhibitory Smads are regulated by ubiquitin-mediated downregulation as a means to control signaling. Based on these backgrounds, Knuesel et al. [36] demonstrated that Smad3 interacts with HSP70 *in vivo* using a tandem affinity purification and mass spectrometry, which might be the first report indicating that Smad3 is a client protein of HSP70 chaperone complex. Smad3 turnover is regulated by the CHIP- (carboxy terminus of Hsp70 interacting protein-) dependent degradation [38]. CHIP is a tetratricopeptide repeat- (TPR-) containing E3 ubiquitin ligase that binds to the molecular chaperones HSC70-HSP70 and HSP90 [39]. Luo et al. [40] analyzed the proteomic profiling of Smad-interacting proteins using mink lung epithelial cells, Mv1Lu cells, and showed that HSP70 is one of interacting partners of Smad2 indicating that HSP70 possibly helps assemble or facilitate the formation of the complex of Smad2 with its partners. Recent studies showed that the chaperon protein HSP90 also positively regulates TGF-*β* receptor activity. TGF-*β* type I and type II receptors were found to interact with and be stabilized by HSP90. Inhibition of HSP90 using small molecule inhibitors leads to increased receptor ubiquitination, indicating that HSP90 is a modifier of Smurf2-mediated TGF-*β* receptor ubiquitination [41]. However, still it is not clear whether HSP90 binding to the receptors depends on and regulates the receptor kinase activity. Since HSP90 functions as part of a multichaperone complex via association with a set of cochaperone proteins that influence the maturation and stability of client proteins [42], we have demonstrated that GA, an inhibitor of HSP90 ATPase, significantly suppresses TGF-*β* signaling. GA-mediated induction of HSP70 interacts with TGF-*β* type I and type II receptors and subsequently degraded TGF-*β* receptors through a proteasome-dependent pathway [43]. When we examined the effects of each component of HSP90 chaperone complex on TGF-*β* signaling using specific siRNAs, HSP70 played a major role in the GA-mediated inhibition of TGF-*β* signaling. Besides chaperoning function of HSPs, numerous reports have shown that the endogenous or stress-induced expression of HSPs, particularly HSP70, confer cellular protection against a variety of stresses and also against physiological stresses associated with growth arrest or receptor-mediated apoptosis. It has been shown that preexposure to heat shock protects TGF-*β* induced apoptosis of cultured hepatocytes with concomitant induction of HSP70 [44]. In addition, GGA attenuated TGF-*β*-induced epithelial-mesenchymal transition (EMT) in renal proximal epithelial cells and enhanced expression of HSP70 using adenoviral infection prevented TGF-*β* induced EMT [45]. Recently, Zhou et al. [46] also demonstrated that HSP70 inhibits EMT by antagonizing the activation and translocation of Smad3 through domain specific interaction with Smad3. To address whether suppression of HSP70 could sensitize the TGF-*β*-induced transcriptional activity and growth arrest in epithelial cell, NCI-H292 cells were transfected with HSP70 siRNA and examined the TGF-*β*-induced reporter activity and cell cycle analysis. As shown in Figure 5, suppression of HSP70 resulted in about 2-fold increase in luciferase activity and the TGF-*β* dependent increase in the percentage of cells accumulating in G_0_/G_1_. Knockdown of HSP70 alone did not lead to increase in G_0_/G_1_ phase compared to control siRNA. To confirm the physiological role of HSP70 in TGF-*β* signaling, we did perform Western blot analyses using lysates from NCI-H292 cells transfected with HSP70 siRNA. The level of endogenous HSP70 expression was significantly reduced that resulted in a decrease in TGF-*β*-induced downregulation of c-Myc. Suppression of HSP70 also resulted in an increase in TGF-*β*-induced phosphorylation of Smad2. These data suggested that endogenous HSP70 acts as a negative modulator in TGF-*β* signaling partially independent of HSP90 activity. However, the exact molecular mechanism underlying the inhibition of TGF-*β* signaling pathway requires further investigation.

## 7. Conclusion

With the discovery of gastric acids and pepsins in the stomach, the questions about “why does the stomach not digest itself in spite of strong lytic weapon?” and “how does the stomach preserve its normal integrity under the continuous exposure to melting materials that are secreted?” had been raised. The discovery of “gastric mucosal barrier” or “stunning defense system” might be the answers to these questions. Secretion into the lumen including bicarbonates, mucus, immunoglobulins, antibacterial substances including lactoferrin, and surface active phospholipids, gastric epithelia, mucosal microcirculation, mucosal immune system, and protective neuron, all these factors are known to contribute to orchestrated artwork of “gastric mucosal protection.” In the recent years, HSPs have been implicated to be an additional factor utilized for the gastric defense mechanisms at the intracellular level. Certain HSPs are expressed even under nonstressful conditions and play an important role in the maintenance of normal cell integrity, but additionally HSPs are generally considered to improve cellular recovery both by either refolding partially damaged functional proteins or increasing delivery of precursor proteins to important organelles such as mitochondria and endoplasmic reticulum, through which HSPs might complete efficient mucosal defense mechanisms and achieve ulcer healing, most probably protecting key enzymes related to cytoprotection, conferring HSPs as “hard worker” for stomach. On the other hand, HSPs can play as immunogen, inflammation hasting, and even carcinogen, simplified as “bad offender” especially in *H. pylori* infection, provoking autoimmune gastritis, atrophy, and cancer promotion. Taken together, HSPs are blamed of unwanted disasters after *H. pylori* infection whereas are awarded by faithfulness and royalty to gastric epithelial cells under the continuous attack of *H. pylori* infection through the achievement of restitution, regeneration, removal of faded cells, and remodeling. Bright hope for therapeutics could be feasible through the modulation of “bad offender” HSPs and enforcement of “hard worker” HSPs in the treatment of gastric diseases.

## Abbreviations

HSP: Heat shock protein
HSF: Heat shock factor
HGP: Human genome project
2D-PAGE: 2-dimentional polyacrylamide gel electrophoresis
SNP: Single nucleotide polymorphism
MAPKK: Mitogen-activated protein kinase kinases
MALT: Mucosa-associated lymphoid tissue.
